# An In Vitro Evaluation of Industrial Hemp Extracts Against the Phytopathogenic Bacteria *Erwinia carotovora*, *Pseudomonas syringae* pv. *tomato*, and *Pseudomonas syringae* pv. *tabaci*

**DOI:** 10.3390/molecules29245902

**Published:** 2024-12-13

**Authors:** Getrude G. Kanyairita, Desmond G. Mortley, Willard E. Collier, Sheritta Fagbodun, Jamila M. Mweta, Hilarie Uwamahoro, Le’Shaun T. Dowell, Mwamba F. Mukuka

**Affiliations:** 1 Department of Agriculture and Environmental Sciences, Tuskegee University, Tuskegee, AL 36088, USA; gkanyairita5762@tuskegee.edu (G.G.K.); dmortley@tuskegee.edu (D.G.M.); jmweta@tuskegee.edu (J.M.M.); 2 Department of Chemistry, Tuskegee University, Tuskegee, AL 36088, USA; huwamahoro5872@tuskegee.edu; 3 Department of Crop Science & Beekeeping Technology, University of Dar es Salaam, Dar es Salaam P.O. Box 35091, Tanzania; 4 Department of Biology, Tuskegee University, Tuskegee, AL 36088, USA; sfagbodun@tuskegee.edu; 5 Department of Mathematics & Computer Science, Alabama State University, Montgomery, AL 36104, USA; ldowell7648@myasu.alasu.edu (L.T.D.); mmukuka1872@myasu.alasu.edu (M.F.M.)

**Keywords:** hemp extract, Erwinia carotovora, *Pseudomonas syringae* pv*. tomato*, *Pseudomonas syringae* pv*. tabaci*, antimicrobial, minimum inhibitory concentration, non-inhibitory concentration, time-kill assay, inhibition zone

## Abstract

Pests and diseases have caused significant problems since the domestication of crops, resulting in economic loss and hunger. To overcome these problems, synthetic pesticides were developed to control pests; however, there are significant detrimental side effects of synthetic pesticides on the environment and human health. There is an urgent need to develop safer and more sustainable pesticides. Industrial hemp is a reservoir of compounds that could potentially replace some synthetic bactericides, fungicides, and insecticides. We determined the efficacy of industrial hemp extracts against *Pseudomonas syringae* pv. *tabaci* (PSTA), *Pseudomonas syringae* pv. *tomato* (PSTO), and *Erwinia carotovora* (EC). The study revealed a minimum inhibitory concentration (MIC) of 2.05 mg/mL and a non-inhibitory concentration (NIC) of 1.2 mg/mL for PSTA, an MIC of 5.7 mg/mL and NIC of 0.66 mg/mL for PSTO, and an MIC of 12.04 mg/mL and NIC of 5.4 mg/mL for EC. Time-kill assays indicated the regrowth of *E. carotovora* at 4 × MIC after 15 h and *P. syringae* pv. *tomato* at 2 × MIC after 20 h; however, *P. syringae* pv. *tabaci* had no regrowth. The susceptibility of test bacteria to hemp extract can be ordered from the most susceptible to the least susceptible, as follows: *P. syringae* pv. *tabaci* > *P. syringae* pv. *tomato* > *E. carotovora*. Overall, the data indicate hemp extract is a potential source of sustainable and safe biopesticides against these major plant pathogens.

## 1. Introduction

*E. carotovora* is a destructive pathogen that causes soft rot and blackleg diseases in a variety of crops, from vegetables to ornamentals and some trees. The bacterium targets tender tissues of storage organs, causing a 40–80% loss during transit, storage, or marketing [1,2,3]. *P. syringae* pv*. tomato* causes bacterial specks in tomatoes [4], produces lesions in leaves and fruits, affects the photosynthetic process, and reduces the overall quality of fruits [5,6]. The bacterium inhibits the growth of most organs, reduces seed yield, and stunts plant height, resulting in varying degrees of yield reduction, ranging from 4 to 40% [7]. *P.syringae* pv. *tabaci* causes not only tobacco diseases but also diseases such as leaf spot in major crops including coffee, soybeans, mung beans, and papaya [8]. These three bacteria are of major economic importance, as they cause significant crop losses worldwide. Currently, there are no known compounds that can remove these bacteria from infected plants. Numerous chemical compounds, including antibiotics, inorganic salts, organic salts, and their combinations, have been evaluated for their effectiveness in mitigating these bacteria. However, they all have significant drawbacks including bacteria developing resistance to the antibiotics and the accumulation of metals from the inorganic and organic salts in soils [9].

Currently, copper-based bactericides are used to impede the proliferation of these pathogens, but resistant strains have developed [10,11]. Furthermore, since copper does not biodegrade, its prolonged use leads to its accumulation in soil [12]. This accumulation can be toxic to soil microorganisms and plants, reducing biological activities and causing soil infertility [13]. Furthermore, the use of non-copper-based synthetic pesticides for the control of plant bacteria is not conducive to sustainable agriculture due to their adverse effects on the environment and public health [12,14].

There is an urgent need to develop safe, affordable, and effective natural biopesticides, and medicinal plants are a huge reservoir of these potential biopesticides. Industrial hemp, which contains phytocompounds such as cannabinoids, terpenes, flavonoids, and alkaloids, has been used medicinally for its anti-inflammatory, antimicrobial, and antifungal properties [15,16,17]. However, little is known about its ability to combat plant pathogens.

Cannabis essential oils contain sesquiterpenes such as nerolidol, caryophyllene, and caryophyllene oxide, along with monoterpenes such as pinene, linalool, and limonene; all known for their antimicrobial properties [18]. Pinene inhibits bacterial growth, myrcene disrupts cell membranes, terpineol and linalool have antibacterial activity, limonene exhibits bactericidal and antibiofilm properties, and nerolidol inhibits the growth of various parasites [19,20,21,22]. Caryophyllene, with antioxidant and anti-inflammatory properties, demonstrates antimicrobial activity without cytotoxicity [23,24].

Although various studies have been conducted to determine the antimicrobial activities of industrial hemp phytocompounds against human pathogenic bacteria, very few have examined antimicrobial activities against plant pathogens. Novak et al. [25] explored the antibacterial properties of essential oils derived from five different cultivars of *Cannabis sativa* L. containing compounds such as pinene, myrcene, trans-ocimene, terpinolene, trans-caryophyllene, and humulene, with undetectable or extremely low levels of delta-9-tetrahydrocsaannabinol (THC) and other cannabinoids. The study revealed antibacterial activity against some common bacteria, including *Staphylococcus aureus*, *Acinetobacter calcoaceticus*, *Beneckea natriegens*, and *Brochothrix thermosphacta* in all five oils [25].

Nissen et al. [26] discovered antibacterial effects in essential oils of *Cannabis sativa* L. seeds against Gram-positive bacteria such as *Enterococcus faecium* and *Streptococcus salivarius* at concentrations below 1% (*v*/*v*). However, yeast, which was included in the same study, was unaffected. Zengin et al. [27] identified the antioxidant properties of hemp essential oils, which show significant antibacterial activity against clinical strains of *Helicobacter pylori*. The MIC for hemp essential oil was 16–64 µg/mL for *Helicobacter pylori* and 8 mg/mL for *Staphylococcus aureus*.

Pellegrini et al. reported antibacterial activity in the essential oil of Futura 75 inflorescences, a variety of *Cannabis sativa* L., against *Staphylococcus aureus* and *Listeria monocytogenes*, with an MIC ranging from 1.25–5 µg/mL but ineffective against *Salmonella enterica*. The oil also exhibited antioxidative properties. In addition, insecticidal activity was observed for some selected insects and they proposed that the insecticidal effect could be attributed to the inhibition of acetylcholinesterase [28].

A study comparing the antimicrobial properties of essential oils from wild hemp inflorescences harvested from northeast Serbia and EU-registered cultivars also highlighted the wide range of CBD concentrations in these wild hemp essential oils (6.9–52.4%) [29]. The wild hemp essential oils assayed showed superior antimicrobial activity against pathogens, including *Staphylococcus aureus*, *Enterococcus faecalis*, *Streptococcus pneumoniae*, *Pseudomonas aeruginosa*, *Yersenia enterocolitica*, *Salmonella enterica*, *Candida albicans*, *Candida krusei*, and *Candida tropicalis* [29]. Moreover, Naveed et al. [30] reported the antimicrobial activities of the ethanol extracts of hemp leaf against several pathogenic bacteria using the inhibition zone assay, showing the possibility of using leaf extracts in place of the more commonly used inflorescence extracts controlling bacteria. To our knowledge, none of the studies investigating the antimicrobial activity of hemp extracts reported their activity against the plant pathogens *E. carotovora*, *P. syringae* pv. *tomato*, and *P. syringae* pv. *tabaci*.

The great variability in the composition of hemp essential oils probably contributes to contradictory results with respect to antimicrobial activity against the same microbial species. This points to a knowledge gap in understanding the synergistic effects of phytocompounds in hemp extracts on antimicrobial activities. Another glaring knowledge gap is the paucity of data on the antimicrobial activity of hemp extracts against plant pathogens. Therefore, more research is needed on the antimicrobial activities of industrial hemp extracts against plant pathogens to improve our understanding of the antimicrobial activities of industrial hemp phytocompounds and their potential for commercial use.

## 2. Results

### 2.1. Industrial Hemp Extract Yield and Cannabinoid and Terpene Metabolomics Analysis

The ethanol extract of the industrial hemp inflorescence had a total yield of 20% calculated using the mass of the dried inflorescences and the mass of the extract after solvent removal. The hemp extract had been characterized in a previous study by a targeted metabolomics method using both LC/MS and GC/MS. Selected data, for compounds present with a concentration greater than or equal to 0.01% (*w*/*v*), are shown in Table 1. More detailed characterization data are in our previous work which compared the extraction efficiency of different organic solvents and deep eutectic solvents in extracting cannabinoids and terpenes from two hemp varieties, citrus and cherry dwarf. Among the organic solvents, ethanol extracted the highest number of compounds, and, among the two varieties, cherry dwarf extracted the highest number. Therefore, ethanol as the solvent and cherry dwarf as the variety were selected to produce the extracts for the current antimicrobial study. The table shows the cannabinoids identified in the extracts and their respective concentration (% *w*/*v*) that were included in the targeted metabolomics analysis and six cannabinoids identified in a non-targeted metabolomics analysis. The four major cannabinoids were CBD (0.69%), CBC, CBN, and CBG (if the concentration was not specified, the peak areas were used to approximately rank the contribution). The extract also contains high amounts of neryl acetate (0.167%), nerolidol 1 (0.133%), nerolidol 2 (0.186%), alpha bisabolene (0.081%), alpha caryophyllene (0.061%), and beta caryophyllene (0.046%). All these compounds are known to be antimicrobial; however, none of these have been evaluated for their antimicrobial efficacy against the three phytobacteria in this study.

### 2.2. Extract Inhibition Zone

**Figure 1:**
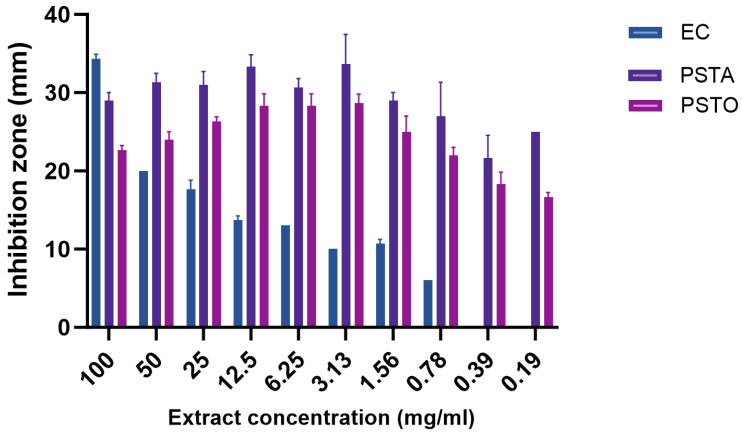
Zones of inhibition at different concentrations of industrial hemp extract against *E. carotovora* (EC), *P. syringae* pv. *tabaci* (PSTA), and *P. syringae* pv. *tomato* (PSTO); significant level observed at *p* = 0.05.

Ten different concentrations (two-fold dilutions 100–0.19 mg/mL) were tested against each of the bacteria *E. carotovora*, *P. syringae* pv*. tabaci*, and *P. syringae* pv. *tomato*, and the results are shown in Figure 1. At the highest concentration (100 mg/mL), the inhibition zone of *E.carotovora* was significantly greater (34.5 mm) than the other two bacteria and its inhibition zone decreased as the concentration decreased to 0.39 mg/mL where there was no inhibition.

*P. syringae* pv. *tabaci* consistently showed large inhibition zones at all concentrations, with the largest zones between 12.5 and 3.13 mg/mL (33 mm). All tested concentrations showed very high potency against *P. syringae* pv. *tabaci*, where even the lowest concentration exhibited a 25 mm inhibition zone Figure 1.

At the highest hemp extract concentration (100 mg/mL), the inhibition zone for *P. syringae* pv. *tomato* was significantly smaller (23 mm) than that of *E. carotovora* and *P. syringae* pv. *tabaci* (29 mm and 34 mm, respectively). Its inhibition zone slightly increased with decreasing concentration to a maximum of 28.7 mm at 3.13 mg/mL, then gradually decreased to 16.7 mm at the lowest concentration (0.19 mg/mL) (Figure 1).

### 2.3. Minimum Inhibitory and Non-Inhibitory Concentration

**Figure 2:**
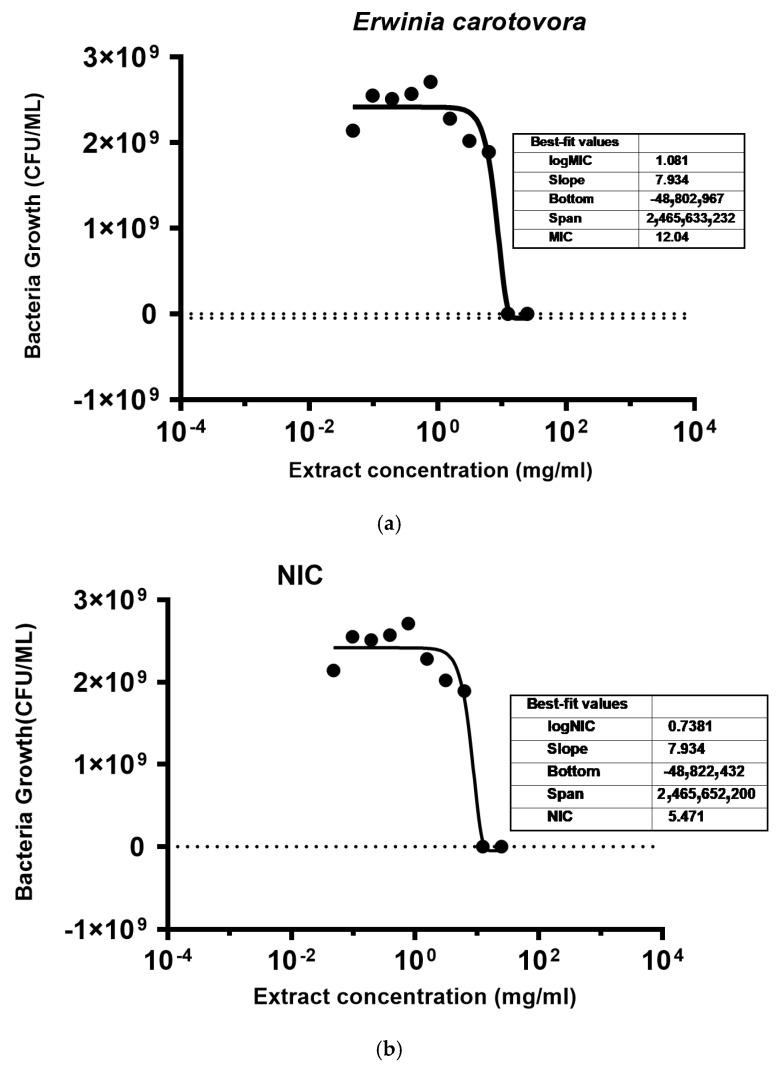
Fitting of the inhibitory effect of hemp extract on *E. carotovora*: (**a**) fit curve showing minimum inhibitory concentration; and (**b**) fit curve showing non inhibitory concentration. Dashed line indicate the baseline concentration at which no more bacteria growth was observed and bullet symbols indicate bacteria concentration.

The Gompertz model [32] was used to predict the minimum inhibitory concentration (MIC) and non-inhibitory concentration (NIC) for the industrial hemp extract against each bacterium. Figure 2 illustrates the fitted curves for the MIC and NIC of the hemp extract against *E. carotovora* and shows the best fit with an R^2^ of 0.96. The Figure 2a curve shows that, among the concentrations studied, 12.04 mg/mL (MIC) is the lowest that can inhibit the growth of *E. carotovora*. Additionally, Figure 2b shows that, below 5.4 mg/mL (NIC), *E. carotovora* continued normal growth.

**Figure 3:**
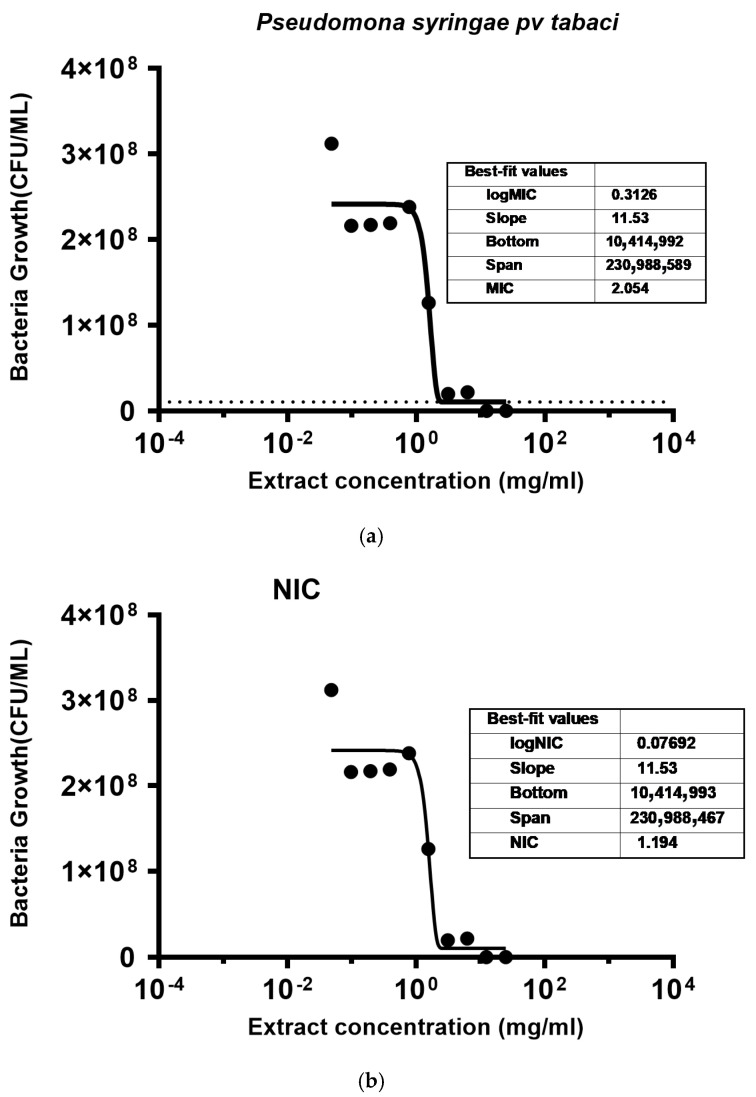
Fitting of the inhibitory effect of hemp extract on *P. syringae* pv. *tabaci*: (**a**) fit curve showing minimum inhibitory concentration; and (**b**) fit curve showing non-inhibitory concentration. Dashed line indicates the baseline concentration at which no more bacteria growth was observed and bullet symbols indicate bacteria concentration.

The fitted curves for the MIC and NIC values of the hemp extract against *P. syringae* pv. *tabaci* are shown in Figure 3. The model predicted an MIC of 2.05 mg/mL and an NIC of 1.2 mg/mL with the coefficient of determination (R^2^) equal to 0.94.

**Figure 4:**
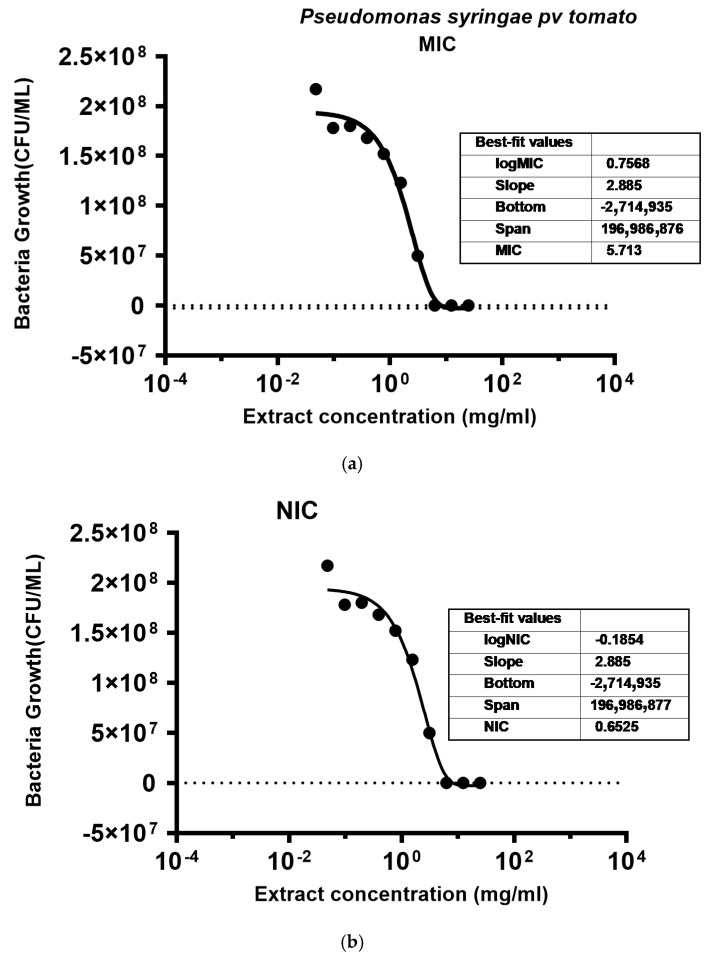
Fitting of the inhibitory effect of hemp extract on *P. syringae* pv. *tomato*: (**a**) fit curve showing minimum inhibitory concentration; and (**b**) fit curve showing non-inhibitory concentration. Dashed line indicate the baseline concentration at which no more bacteria growth was observed and bullet symbols indicate bacteria concentration.

The fitted curves for both MIC and NIC values of *P. syringae* pv. *tomato* are shown in Figure 4. The model predicted an MIC of 5.7 mg/mL and an NIC of 0.65 mg/mL with a coefficient of determination (R^2^) equal to 0.98.

### 2.4. Growth Reduction

**Figure 5:**
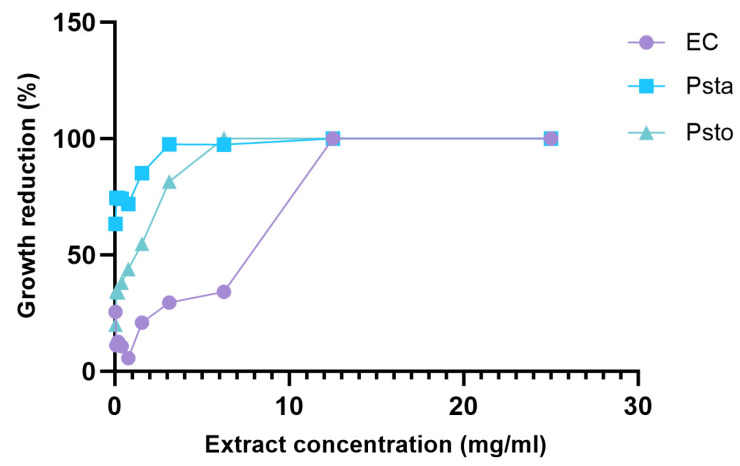
Percentage growth reduction in *E. carotovora* (EC), *P. syringae* pv. *tabaci* (PSTA), and *P. syringae* pv. *tomato* (PSTO) at different hemp extract concentrations.

The percentage growth reduction in different hemp extract concentrations for each of the bacteria is shown in Figure 5. The reduction in *E. carotovora* growth ranged from 100% to 5.7% depending on the hemp extract concentration used. The two highest concentrations (25 and 12.5 mg/mL) inhibited bacteria growth by 100%; however, all other concentrations inhibited growth by less than 50%.

The hemp extract had a percentage growth reduction on *P. syringae* pv. *tabaci* ranging from 100% to 63.3%. The first two concentrations (25 and 12.5 mg/mL) reduced growth by 100%, 6.25 and 3.13 mg/mL had a 97% growth reduction, and the lowest concentration 0.038 mg/mL reduced growth by 63.3%.

Three hemp extract concentrations (25, 12.5, and 6.25 mg/mL) reduced the growth of *P. syringae* pv. *tomato* by 100%. The next highest growth reduction was from 3.13 mg/mL (81.6%), while the two lowest hemp extract concentrations (0.075 and 0.038 mg/mL) inhibited growth by 54 and 20%, respectively.

Overall, the hemp extract concentrations showed the highest growth reduction in *P. syringae* pv. *tabaci* compared to the other two plant pathogens studied. Furthermore, the hemp extract concentrations that showed high potency were between 25–3.13 mg/mL for both *P. syringae* and 25–12.5 mg/mL for *E. carotovora*.

### 2.5. Time-Kill Assay

**Figure 6:**
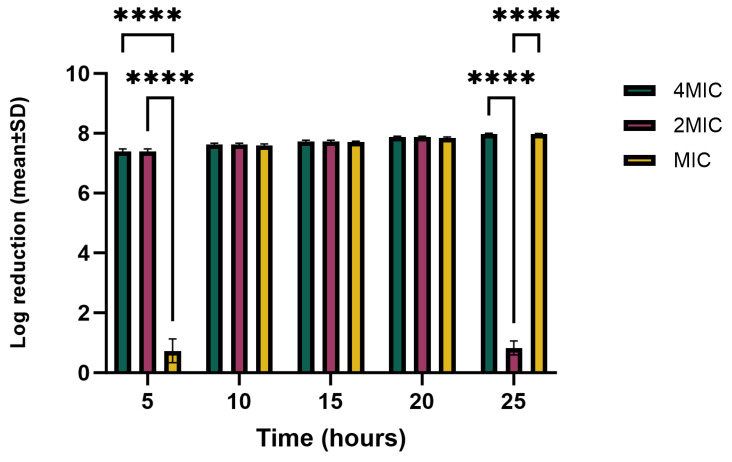
Log_10_ reductions in *P. syringae* pv. *tomato* CFU at incubation time intervals and MIC, 2 × MIC, and 4 × MIC concentrations. Tukey’s multiple-comparison post hoc test showed significant statistical differences (**** *p* < 0.0001) between the indicated data.

A time-kill assay was conducted using hemp extract concentrations of MIC, 2 × MIC, and 4 × MIC, and the activity of each bacterium was determined at different time intervals during the growth of the bacteria. The log_10_ reduction for *P. syringae* pv. *tomato* is shown in Figure 6. During the first five hours, the activity differed significantly between the MIC and both 2 × MIC and 4 × MIC (*p* < 0.0001); however, there was no significant difference in activity between 2 × MIC and 4 × MIC. During this time interval, both 2 × MIC and 4 × MIC reduced the growth of the bacteria by 99%, while there was only a slight decrease in colony-forming unit (CFUs) with the MIC. Over the 10 to 20 h time interval for all concentrations, the activity against the three bacteria was very high and their growth was reduced by approximately 99%. From 21 h to when the experiment was terminated after 24 h, 4 × MIC and MIC continued to show a growth reduction of almost the same magnitude; however, during the last time interval, 4 × MIC and 2 × MIC differed significantly (*p* < 0.0001), which is attributed to the regrowth of bacteria in the 2 × MIC. Moreover, there was a significant difference between 2 × MIC and MIC (*p* < 0.0001), probably due to bacteria regrowth in the 2 × MIC. The findings show that the MIC starts to inhibit bacteria after 5 h of exposure and remained steady until the end of the growth period. Further investigation is required to determine why there was a regrowth of bacteria during the last phase with 2 × MIC.

**Figure 7:**
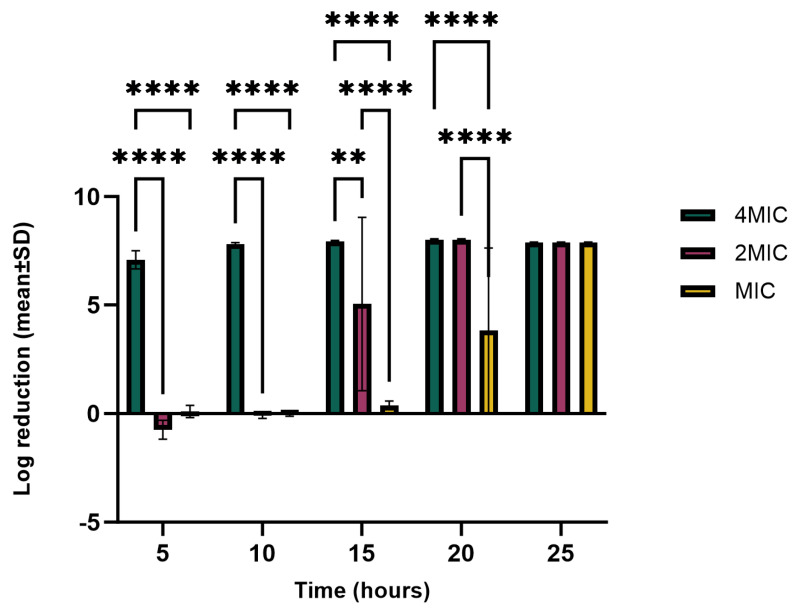
Log_10_ reductions in *P. syringae* pv. *tabaci* CFU at incubation time intervals and MIC, 2 × MIC, and 4 × MIC concentrations. Tukey’s multiple-comparison post hoc test showed significant statistical differences (** *p* < 0.01, and **** *p* < 0.0001) between the indicated data.

The reduction in the growth of *P. syringae* pv. *tabaci* at different time intervals when exposed to the hemp extract concentrations of 4 × MIC, 2 × MIC, and MIC is shown in Figure 7; 4 × MIC reduced growth by 99% and differed significantly from both 2 × MIC and MIC (*p* < 0.0001) during most of the assay’s time interval (0–21 h). By the last time interval (21–24 h), there was no significant difference between the log_10_ reduction in MIC, 2 × MIC, and 4 × MIC. During the first 5 h, there was no significant difference between 2 × MIC and MIC, as both had no significant growth reduction. A similar condition was observed after 10 h of exposure, where there was no significant growth reduction with 2 × MIC and MIC. After 15 h of exposure to the hemp extract, a growth reduction was observed with 2 × MIC which was significantly different from 4 × MIC (*p* < 0.001) (two-fold difference in reduction) and MIC (six-fold difference in reduction) (*p* < 0.0001). Although the MIC started to show some inhibition after 15 h, a significant difference was still observed between MIC and both 2 × MIC and 4 × MIC (*p* < 0.001). After 20 h, the growth reduction in 4 × MIC and 2 × MIC were not significantly different but both were different from that of the MIC (four-fold difference in reduction) (*p* < 0.0001). During this period, the growth reduction with the MIC was above 90%, while both the 4 × MIC and 2 × MIC growth reduction were above 99%. Generally, 4 × MIC reduced the growth of the bacteria from the first 5 h and was able to maintain its potency throughout the growth period; 2 × MIC was able to achieve its maximum growth reduction after 24 h, although, after 15 h, the reduction was approximately 90%. At the end of the bacteria growth period, MIC had achieved its highest growth reduction with a peak time of twenty hours.

**Figure 8:**
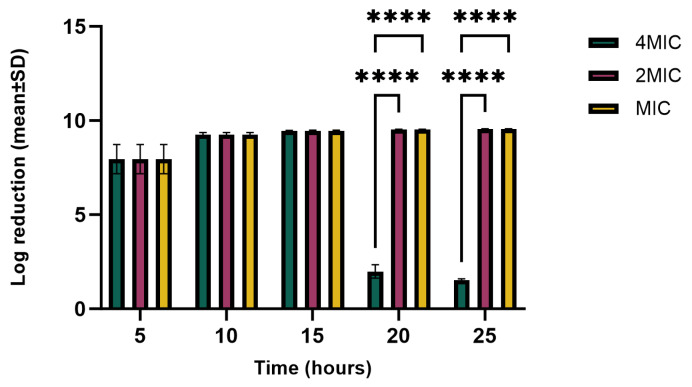
Log_10_ reductions in *E. carotovora* CFU at incubation times intervals and MIC, 2 × MIC, and 4 × MIC concentrations. Tukey’s multiple-comparison post hoc test showed significant statistical differences (**** *p* < 0.0001) between the indicated data.

The reduction in *E. carotovora* growth over time intervals is shown in Figure 8. There was no significant difference between 4 × MIC, 2 × MIC, and MIC up to 15 h of hemp extract exposure to bacteria. During the first five hours, the growth reduction was slightly lower than 10–15 h, although the reduction was approximately over 90%. After 15 h, the bacteria in the 4 × MIC treatment started to regrow, reducing log_10_ reduction 8-fold lower than MIC and 2 × MIC throughout the growth period; 2 × MIC and MIC maintained their growth reduction until the end (over 99.99%), which differs significantly from 4 × MIC (*p* < 0.0001). The findings show that MIC and 2 × MIC were able to reduce *E. carotovora* growth throughout the growing period to 99.99%. The reasons for the regrowth of bacteria during 4 × MIC treatment after 20 h need to be investigated.

## 3. Discussion

In this study, we employed three distinct assays: the inhibition zone, minimum inhibitory/non-inhibitory concentration (MIC/NIC), and time-kill assay. These methodologies are indispensable in evaluating antimicrobial efficacy. They provide insights into the potency and mechanisms of action of antimicrobial agents against pathogens. The inhibition zone assay indicates the effectiveness of an antimicrobial agent and susceptibility, while MIC values assist in determining pathogen susceptibility by quantifying the minimal concentration of an antimicrobial required to inhibit visible growth [33]. The NIC values are pivotal in assessing pathogen resistance to antimicrobial agents by identifying the lowest antibiotic concentration that does not inhibit growth, thus facilitating an understanding of the resistance mechanisms involved [34]. The time-kill assay evaluates bactericidal activity over time, elucidating the dynamics of microbial reduction and offering a comprehensive evaluation of antimicrobial efficacy over time [35]. The collective information obtained from these assays complements each other, aiding researchers in identifying effective antibiotics against bacteria while managing antibiotic resistance challenges. Consequently, we believe it is crucial for researchers attempting to identify effective phytocompounds against plant pathogens to implement more than one assay in their studies. Despite the numerous studies investigating the efficacy of plant material extracts against these bacteria, inconsistencies are common in the type of assays conducted, the presentation of concentrations used, and the use of treatment controls [36]. The standardization of protocols is vital for ensuring reliable and reproducible results. Given the absence of literature regarding the efficacy of industrial hemp against the plant pathogens investigated in this study, we focus on discussing the effectiveness of the hemp extract compared to other plant extracts in controlling the investigated plant pathogens. We also highlight the lack of consistent protocols that make direct comparisons essentially impossible. Recent studies selected for comparison were randomly chosen and are not exhaustive.

The highest concentration (100 mg/mL) inhibition zone for *E. carotovora* was 34 mm; this zone was much larger than the one observed in a study by Hamad et al. [37] of *E. carotovora* using 100% concentrated aqueous extracts of *A. herba alba* (13.33 mm), *Pistacia atlantica* (12 mm), and *Juniper phoenicia* (11.67 mm). In another study by Vasinausikiene et al. on the activity of essential oils against *E. carotovora* using the disc diffusion method, the results revealed no activity from the essential oils studied except for the oils of *Origanum vulgare*, which showed an inhibition zone between 2–6 mm [38]. All these reported inhibition zones are smaller than our findings of 34 mm against *E. carotovora*. While these studies seem to indicate the industrial hemp extract is more effective against *E. carotovora* than these other plant extracts, any comparisons between these studies should be evaluated cautiously as these studies cannot be easily compared because of the difference in plant species studied, different assay methods, and different concentrations used in each study. However, the inhibition zone assay does indicate there are compounds in the industrial hemp extract that are highly active against *E. carotovora* either as a single compound or synergistically among several.

In the experiment on *P. syringae* pv. *tomato* and *P.s syringae* pv. *tabaci* the highest zone of inhibition was 28 mm (12.5 mg/mL–3.13 mg/mL) and 33 mm (12.5 mg/mL–3.13 mg/mL), respectively. These findings are similar to those obtained by Elkhalfi et al. [39] with *Aizoon canariense* (20 mm), *Geranuim robertianum* (30 mm), and Rubia peregrina (18 mm) against *Pseudomonas syringae* pv. *tomato*. Furthermore, our results were in the range of those obtained by Arasu et al. [40], where the *Albizia lebbeck* ethyl acetate extract inhibition zone against *P. syringae* pv. *tomato* was 24 mm. The hemp extract in our findings mirrored the findings with *Thymus migricus* (30 mm) and *Mentha longifolia* (16 mm) [41]. However, our inhibition zones were much larger than those of Garcia-Latore et al. [42], where a 0.39 mm zone was obtained when an *Alternaria leptinellae* E138 extract was assayed.

The MIC of the hemp extract against *E. carotovora* was 12.04 mg/mL, and was comparable to the MIC (12,500 µg/mL) reported by Saadoun et al. [43] for the extract of *Orobanche cernua* against *E. carotovora*. Saadoun et al. also studied other species of Orobanche and found even higher MICs (27,000 µg/mL and 63,000 µg/mL). In our study, although the MIC was determined to be 12.04 mg/mL, further analysis was performed to determine the ability of other concentrations to inhibit bacteria growth. The highest concentrations (25 and 12.5 mg/mL) could completely inhibit bacteria growth, achieving 100% performance; the other remaining concentrations only achieved an inhibition of 5–34%.

The MIC for *P. syringae* pv. *tomato* and *P. syringae* pv. *tabaci* growth were 5.7 mg/mL and 2.05 mg/mL, respectively. These findings are similar to Elkhalfi et al. [39], who obtained an MIC of 2.6 to 4 mg/mL when three potent plant extracts were tested against *P. syringae* pv. *tomato*. Our results were around an order of magnitude of those reported by Arasu et al. [40], who reported an MIC of 150 µg/mL against PSTO; Sanchez-Hernandez et al., who reported an MIC of 750 µg/mL against *Pseudomonas* spp. [44]; and Garcia-Latorre, who reported an MIC of 300 µg/mL against PSTO [42]. The MIC results obtained from our study of both bacteria are lower than the MIC of 30 g/L obtained in the experiment conducted by Pinto et al. [45] using the aqueous extract of eucalyptus leaves. In the same study, 29 g/L was found to be the concentration that inhibited the growth of 50% of the bacteria population; however, our findings revealed that 1.56 mg/mL and 0.04 mg/mL inhibited the growth of 50% of PSTO and PSTA, respectively.

Our study calculated an NIC of 0.65 mg/mL for PSTO, an NIC of 1.2 mg/mL for PSTA, and an NIC of 5.5 mg/mL for EC. These are the highest concentrations at which pathogens are not inhibited, revealing insights into their resistance profiles [34]. An extended exposure of bacteria to concentrations equal to or below NIC values acts as selective pressure, facilitating the survival and predominance of bacteria possessing mutations or resistance mechanisms [46]. Furthermore, the exposure may augment the probability of mutations, some of which could impart resistance to increased antibiotic concentrations [46]. None of the reviewed studies reported the NIC; therefore, we were unable to compare our findings with others.

To further investigate the susceptibility of test bacteria to hemp extract, the MIC results were used in a time-kill assay to determine how long it takes to inhibit the growth of bacteria and to predict the mode of action, whether the hemp extract kills bacteria or delays its growth. The results show that the MIC (12.04 mg/mL) was able to inhibit *E. carotovora* growth throughout the growing period (24 h), with an over 99% inhibition. A study by Charirak et al. [47] obtained similar findings with *Piper betle* extract. In their study, the 4 × MIC achieved the complete inhibition of growth in 6 to 8 h, similar to our findings of 5 h. In contrast, Malvin Joe et al. [48] found that green tea extract killed *E. carotovora* after 18 h using 10×MIC (MIC = 7 mg/mL).

We were unable to find any article that performed time-kill assays on *P. syringae* pv. *tomato* and *P. syringae* pv. *tabaci* that we could use to compare with our results. Our *P. syringae* pv. *tomato* time-kill assay showed susceptibility to MIC and 4 × MIC throughout the growing period with an inhibition of 99.99%. *P. syringae* pv. *tabaci* was the most susceptible to all concentrations throughout the all-time intervals, inhibited to 99.99% by 20 h of the experiment. It was observed from our results that the MIC of all the studied bacteria inhibited growth by 99.99% by the end of the experiment. In contrast, 2 × MIC inhibited the growth of EC and PSTA, while it slowed the growth of PSTA for 21 h; 4 × MIC inhibited the growth of PSTA and PSTO while it slowed the growth of EC for 15 h. As mentioned above, the time-kill assay can be used to predict the mode of action of a bactericide. The results discussed above indicate that the mode of action of the hemp extract against *E. carotovora* and *P. syringae* pv. *tomato* is a combination of killing and slowing growth, while, in *P. syringae* pv. *tabaci*, the predominant mode of action is killing.

Since *P. syringae* pv. *tomato* and *E. carotovora* showed the regrowth of bacteria at 2 × MIC after 20 h and 4 × MIC after 15 h, respectively, this points to stronger defence mechanisms against the hemp extract. The regrowth of Gram-negative bacteria under attack by an antimicrobial agent can be attributed to two mechanisms triggered by quorum sensing (QS) that defend against the antimicrobial agent. QS is bacterial communication, especially under a high population density [49], that regulates bacteria growth by turning on the battery survival gene [50]. The battery survival gene regulates biofilm formation and the efflux pump [50]. Biofilm formation occurs when bacteria form aggregates that are enclosed in a matrix of extracellular polymeric substances (EPSs), which can hinder the penetration of antimicrobial agents and protect the inner cells [51]. Biofilm formation increases the chances of survival of bacteria by prolonging their life so that, even if the outer layers of the biofilm are killed, the inner cells may survive and later regrow and colonize [52]. The biofilm is made up of exopolysaccharides, proteins, nucleic acids, and lipids, which are released by the bacterium [52]. When the biofilm is fully made and mature, the new colonies regrow and colonization begins again. Since the MIC of the hemp extract did not show any regrowth in any of the studied bacteria, it seems to indicate that, if the hemp extract is used at this concentration, the battery survival gene will not be activated in these bacteria. However, additional studies are needed to investigate the effect of the hemp extract on quorum sensing, battery survival gene activation, biofilm formation, and efflux pump triggering. All these areas are under active investigation for plant pathogenic bacteria, as exemplified by the active research effort searching for plant-based compounds that can inhibit biofilm formation. For example, Qi et al. [53] found that *Rheum tanguticum* was able to inhibit the virulence of *Pectobacterium carotovorum* by inhibiting the beta lamactase enzyme that causes resistance in the bacterium. However, the research effort for human pathogenic bacteria far exceeds that for plant pathogenic bacteria. Therefore, there is a huge knowledge gap in the current understanding of bacteria defence mechanisms such as biofilm formation, the mechanism of bacterial regrowth at high concentrations of antibacterial agents, signaling pathways, and enzymes involved for plant pathogenic bacteria.

## 4. Materials and Methods

1. Sources of Plant Materials and Microorganisms

*Cannabis sativa* L. cherry dwarf (industrial hemp) variety flowers were harvested from the George Washington Carver Agricultural Experiment Station at Tuskegee University, latitude 32°26′11.045″ N and longitude −85°44′11.388″ W, air-dried, and stored. *E. carotovora* was purchased from Carolina Biology, while *P. syringae* pv. *tomato* and *P. syringae* pv. *tabaci* were obtained from Utah State University.

2. Extraction of Plant Material

The extraction procedure explained in Kanyairita et al. [31] was adopted as follows: Air-dried industrial hemp flowers were ground using a heavy-duty laboratory grinder and stored in amber containers. An accurate weight sample (20 g) of inflorescence powder was weighed and placed in a 1 L flask, followed by addition of 400 mL of ethanol. The flask was placed in a water shaker bath set at 37 °C, and 75 rpm for 24 h. The hemp extract was filtered using Whatman No. 1 filter paper to separate the plant material from the ethanol extract.

3. Extract Cleaning and Homogenization

The ethanol extract was defatted to remove waxes and lipids. The extract was placed in a freezer (−20 °C) for 72 h, followed by vacuum filtration. To remove chlorophyll, the extract was then mixed with activated carbon in a 10:1 (mL/g) ratio with gentle shaking for 3 h. The mixture was vacuum-filtered using Whatman No. 2 filter paper, followed by a 0.22 µm syringae filter. The filtered hemp extract was dried using a rotary evaporator, and the yield was determined. The extract was homogenized using a Fisherband Model CL-18 sonicator set at 50% amplitude and pulsed at 3 s on and 5 s off, for 2 min. To make a 200 mg/mL stock solution, 2.00 g of hemp extract was dissolved in 10.0 mL of 99.9% DMSO.

4. Inhibition Zone Assay

The first industrial hemp extract antimicrobial activity experiment was an inhibition zone assay using the agar well diffusion method by Nigussie et al. with some modifications [54]. From the stock solution, a series of working concentrations were prepared by two-fold dilutions with 5% DMSO to make a total of ten concentrations ranging from 100 mg/mL to 0.156 mg/mL. Bacteria were grown using nutrient broth (EC) and Lysogeny broth (LB) (PSTO and PSTA) at 37 °C and 28 °C, respectively. The overnight culture was centrifuged for 5 min at 4900 rpm.

Broth was decanted, with the culture resuspended in new broth; it was vortexed, and the absorbance determined. The culture was used in the subsequent experiment if the absorbance was greater than 0.5 at 600 nm. The culture was diluted with PBS to 1 × 10^6^ CFU/mL (by matching with 0.5 McFarland standard) and seeded on agar plates. Using a sterilized 8 mm corkscrew, holes were made in the agar plate, followed by pipetting 100 µL of hemp extract into the holes. The extract was allowed to percolate inside the agar and dried under the laminar flow hood. Plates were incubated at their respective temperature requirements overnight. The zone of inhibition was measured for each concentration using a vernier caliper. For each concentration, the procedure was replicated on three different plates three different times to validate the results.

5. Minimum Inhibitory and Non-Inhibitory Concentrations Assays

The minimum inhibitory concentration was determined for each bacterium using three separate 96-well microtiter plates as follows: A plate was labeled from left to right, with each column representing one concentration and the last two columns as positive and negative controls. One hundred microliters (100 µL) of broth were added to each well. In the first well of each row, 100 µL of a 100 mg/mL hemp extract solution was added and mixed by pipetting in and out. Then, 100 µL was withdrawn from the first well and added to the next well in the same row and mixed serially until the 10th column wells. The 11th column wells were used as a positive control (broth and bacteria) and the 12th column wells were used as a negative control (broth only), so no hemp extract was added to these wells. From the 10th well, 100 µL was withdrawn and discarded to maintain uniform volume in all wells. The serial dilutions were repeated in five rows on this 96-well plate; the last three rows were used as a control of the extract, as only the extract solutions without bacteria were added [43,44]. Thereafter, 50 µL of 1 × 10^6^ CFU/mL bacteria culture was added to all wells except the negative and extract control wells [55]. The plate was incubated for 18 h at the temperature specified for each bacterium. For visual determination of MIC, 20 µL of 3-(4,5-dimethyl-2-thiazolyl)-2,5-diphenyl-2H-tetrazolium bromide (MTT) was added in all wells and the color change was observed. Purple indicates growth, while yellow indicated no growth [55]. The minimal inhibitory concentration was the lowest concentration at which no observable growth was observed after the addition of MTT [55]. The experiment was carried out in triplicate.

To validate the results, another experiment was performed in which the bacteria culture grown overnight was serially diluted (ten-fold); the optical density (600 nm) was recorded for each serial dilution using the Accuris microplate reader (MR9600). From each serial dilution, a 100 µL aliquot was taken and seeded on an agar plate, then incubated at 37 °C for 24 h. Colonies were counted on plates with colony counts between 30–300 and the CFU/mL for the original culture was determined using Equation (1). This information was used to construct the calibration curve for the subsequent procedure. The optical density (OD) of each well on a 96-well plate from the previous MIC experiment before adding MTT was determined using an Accuris microplate reader (MR9600), while the CFU/mL of each well was determined by inserting the OD into the equation generated from the standard curve information. Quantitative MIC and NIC were determined using the Gompertz model in GraphPad Prism 10.

(1) $$\frac{cfu}{ml}=\left(number\;of\;colonies\;counted\times dilution\right)÷Volume\;of\;sample\;plated\;\left(ml\right)$$

6. Time-Kill Assay

For each bacterium, its minimum inhibitory concentration (MIC) was used to prepare single (MIC), double (2 × MIC), and quadruple (4 × MIC) concentrations. The time-kill assay was prepared in a 96-well plate, with Phyton 35 as a positive control following manufacturer’s recommended rate of 1.56 mL [56]. The plate was incubated inside the plate reader with programmed kinetic readings. The optical density obtained at each time interval was used to determine CFU/mL. The logarithmic reduction was calculated using Equation (2) at each time interval, where A is the CFU for the control (Phyton 35) and B is the CFU for the test concentrations. The data from the time-kill assay were analyzed using ANOVA in GraphPad Prism 10 [56,57].

(2) $$\log_{10}A-\log_{10}B$$

## 5. Conclusions

The data show the susceptibility of the test bacteria to hemp extract, ordered from the most susceptible to the least susceptible, as follows: *P. syringae* pv. *tabaci* > *P. syringae* pv. *tomato* > *E. carotovora*. This study indicates that hemp extract is effective in controlling *E. carotovora*, *P. syringae* pv. *tabaci*, and *P. syringae* pv. *tomato*. However, the hemp extract is more effective against both *Pseudomonas* spp. than *E. carotovora*. The difference could be due to their difference in cell wall structure, resistance mechanisms, and metabolic pathways. More studies are needed to determine how hemp extract causes stress to bacteria such as interference with quorum sensing, biofilm formation, and oxidative stress. Moreover, to ensure sustainable agricultural practices that are safe and affordable for low-income farmers, synergistic effect studies are needed between hemp compounds in the extract and, more importantly, between hemp extracts and other plant extracts.

## Figures and Tables

**Table 1:** Chemical profile of cherry dwarf hemp ethanol extract adapted from Kanyairita et al. [31]. The amount is shown as mean ± SD and NS indicates Not Specified.

Compound Name	Compound Type	Type of Analysis	Amount (%)
Cannabidiol	Cannabinoid	Targeted	0.692 ± 0.00
Delta-9-tetrahydrocannabinol	Cannabinoid	Targeted	0.196 ± 0.00
Nerolidol 2	Terpene	Targeted	0.186 ± 0.015
Neryl acetate	Terpene	Targeted	0.167 ± 0.016
Nerolidol 1	Terpene	Targeted	0.133 ± 0.028
Alpha bisabolene	Terpene	Targeted	0.105 ± 0.010
Cannabinol	Cannabinoid	Targeted	0.082 ± 0.00
Beta bisabolene	Terpene	Targeted	0.081 ± 0.013
Alpha caryophyllene	Terpene	Targeted	0.061 ± 0.018
Beta caryophyllene	Terpene	Targeted	0.046 ± 0.010
D-limonene	Terpene	Targeted	0.045 ± 0.02
Cannabichromevarin	Cannabinoid	Nontargeted	NS
Cannabichromene	Cannabinoid	Nontargeted	NS
Cannabicitran	Cannabinoid	Nontargeted	NS
Cannabidiolic acid	Cannabinoid	Nontargeted	NS
Cannabidivarin	Cannabinoid	Nontargeted	NS
Cannabinodiol	Cannabinoid	Nontargeted	NS

## Data Availability

The data are contained within the article.
